# Multiple sclerosis and pregnancy; What a neurologist may be asked for?

**Published:** 2014-04-03

**Authors:** Bahaadin Siroos, Mohammad Hossein Harirchian

**Affiliations:** ^1^ Department of Neurology, School of Medicine, Imam Khomeini Hospital, Tehran University of Medical Sciences, Tehran, Iran

**Keywords:** Multiple Sclerosis, Pregnancy-related, Immunomodulation, Postpartum Period

## Abstract

Multiple sclerosis (MS) is the most common chronic autoimmune demyelinating disorder of the central nervous system (CNS) which preferentially involves young women in early child bearing age. Opposite to traditional view emphasized on discouraging female patients from enduring pregnancy, recent investigations showed that pregnancy-related physiological alterations, especially during the third trimester, reduce the annual relapse rate of multiple sclerosis up to 80% which is comparable with conventional disease modifying drugs. Nowadays, expert’s viewpoint is that female patients should not be discouraged from having children. Nonetheless, who and when should be allowed to endure gestational period is a complex decision which should be taken for every patient individually. It necessitates that neurologists be aware of updated information regarding pregnancy-related fetal and maternal considerations in patients with MS. In this brief review, it was tried to discuss this topic according to available data and guideline-based recommendations.

## Introduction

Multiple sclerosis (MS) is a chronic autoimmune demyelinating disease with preferential involvement of young women in early child bearing age.^1^ Of interest, recent studies showed that gestational period not only has no harm effect on the long-term course of multiple sclerosis, but also may play as a disease modifying factor particularly during the third stage of pregnancy.^2^ This was in contrary to the traditional view that discouraged MS patients from having children. Thereupon, managing fertile period became a serious concern for patients and their family which necessitate updating our knowledge in this important subject. 

Here, we summarized updated information on the subject of MS and pregnancy according to main articles and guideline-based recommendations published in the English literatures. Based on expert’s viewpoint, our primary aim was to finding out reasonable practical points on important fetal and maternal considerations such as reciprocal interaction of MS and pregnancy, the risk and benefit of continuation or discontinuation of disease-modifying drugs during pregnancy and lactation, and managing of delivery and postpartum period, etc. 

***1.    Do pregnancy-related physiological alterations could have immunomodulatory effect?***

It is well recognized that immunomodulation secondary to pregnancy-related physiological alterations affects the natural course of some autoimmune disorders.^1-2 ^Pregnancy, especially during the third trimester, has been shown to reduce the annual relapse rate of MS up to 80% which is comparable with conventional disease modifying treatments.^3^ Likewise, Ponsonby et al. recently reported that pregnancy may reduce the risk of demyelinating attack.^4^ On the other side, approximately 30% of patients experience at least one relapse in the first three months of post-partum stage.^2^ The precise mechanism of the pregnancy induced immunomodulation is not fully recognized to date. Pregnancy related endocrine immunoregulation has received an increasing attention in the last decade and seems to be contributed in the amelioration of MS activity especially during the third trimester.^5^ In this dissertation, multiple studies showed that pregnancy estrogen trial reduces the activity of cell-mediated diseases such as experimental autoimmune encephalomyelitis (EAE).^6^^-^^7^ Several biological factors including the reduction of adhesion molecules and Th1 inflammatory cytokines (IL-2 and INF-γ), down-regulation of antigen presenting cells associated with up-regulation of Th2 cytokines (IL-4 and IL-10) and regulatory T cells (T-reg cells) had been proposed to be the main cause of immunomodulatory effect of gestational hormones.^5^^,^^8^^-^^14^ Other immune cells especially Th17, and natural killer (NK) cells became the topic of interest so that several investigations have been designed to explore the potential role of them in immunomodulatory alterations of pregnancy compared to non-pregnant patients.^1^^,^^15^^-^^17^ NK cells especially CD56^bright^CD16^-^ subset are the main immune cells in human decidua tissues which play a pivotal role in modulating trophoblast invasion.^1 ^NK cells CD56^bright^ subset has been shown to be upregulated in the third trimester and decreased in postpartum period which may, to some extent, explain the immunomodulatory effects of pregnancy on MS activity.^18^ This is supported by De Jager et al. observations describing the decreased number of CD56^bright^cells in MS patients compared to healthy subjects.^19^ Of interest, disease modifying drugs including interferon-β and the monoclonal antibody, daclizumab, upregulate CD56^bright^subpopulations.^20^

Vitamin D, which in the second and third trimester is about two-fold higher than postpartum stage, has been speculated to be shared in MS remission in gestational period.^21^ Protective effect of vitamin D on MS course was mainly seen in women but not in men.^22^^-^^23^ Thereupon, some researchers suggested that estrogen plays an important role in vitamin D mediated inhibition of demyelinating diseases.^24^

Other than modulation of maternal immune system, there is accumulating evidence that the brain repair capacity and neuroplasticity enhance during pregnancy. Gregg et al.^25^ showed that prolactin augments white matter remyelinization in pregnant mice. Likewise, Franssen et el.^26^ have reported that after a neural insult, neural recovery was significantly higher in multiparous rats compared to the virgins. This is compatible with Ponsonby et al. observations which showed that having one, two, three, four, and five children respectively have been associated with 51%, 61%, 73%, 80%, and 94% reduction in the risk of development of demyelinating disorders.^4^

Overall, it appears that Th2 phenotype shift, upregulation of T-reg cells and CD56^bright ^subpopulations associated with increased neuroplasticity which happen in a setting of hormonal and metabolic alterations during gestational period play main role in pregnancy related MS remission. Elucidating precise mechanism of this phenomenon may shed new light on designing novel therapeutic strategies for this disabling disease.

***2.    Do parents’ MS increase the risk of disease in their children?***

According to available data, lifetime risk of MS in normal population is approximately 100-300 cases per 100,000.^27^ The risk for developing MS increased to 2-4% in persons for whom one of their first relatives is involved, and up to 20% in children for whom their both parents have MS.^28^^,^^29^ Although, family history of disease was reported in about 15% of MS patients, to date, positive family history of disease is considered as a red flag for the diagnosis of MS.^27^ Therefore, according to common sense of experts in this area, it is recommended that we should not exaggerate the risk for developing MS in patient’s children, but from a psychological view, it is better to say that the lifelong probability for having a normal child is approximately as high as 96%.

***3.    Which MS patients are allowed to become pregnant?***

Approximately 33% of MS patients are at the risk of relapse in post partum period.^2^ High disease activity during and one year before pregnancy as well as high expanded disability status scale (EDSS) before gestation increase the risk of relapse.^2^^,^^30^ Therefore, it has been recommended to give permission for pregnancy in patients with no disabling relapse within last year and even no active lesion in recent brain Magnetic Resonance Imaging (MRI). It is clear that patients with high EDSS would have a high risk pregnancy and is better to be discouraged about child bearing.

***4.    Should disease modifying drugs be discontinued before pregnancy?***

Available data about MS disease modifying FDA-approved drugs is mainly based on animal research or incidental exposure to drug, which cannot simply generalize them to human as high evidence recommendation (Table 1).^31^^-^^32^ The expert’s recommendation is to stop disease modifying drugs (DMDs) prior to conception.^33^

*Interferon-β (INF-β):* According to a recent systematic review, INF-β exposure has been associated with lower mean birth weight, shorter mean birth length, and preterm birth but not associated with congenital anomaly, spontaneous abortion, or cesarean delivery.^34^ Recently, Amato et al.^35^

**Table 1:** Risk category classification of MS drugs in pregnancy and lactation

**Modifying drug**	**Common Indication**	**FDA category in pregnancy**	**Risk category in lactation**
INF-β-1a	RRMS*	C	L3
INF-β-1b	RRMS	C	L3
Glatiramer acetate	RRMS	B (Safest)	L3
Fingolimod	RRMS	C	Unknown
Natalizumab	PRMS*	C	Unknown
Teriflunomide	PRMS	X (Contraindicated)	Unknown (Contraindicated)
Mitoxantrone	SPMS*, PRMS	D (Teratogen)	L5 (Contraindicated)

Based on reference 27 modified from Houtchens.^31^

* RRMS: Relapsing remitting multiple sclerosis, PRMS: Progressive relapsing multiple sclerosis, SPMS: Secondary progressive multiple sclerosis

investigated fetal and maternal outcome of 388 pregnant women who incidentally had been received INF-β within four weeks of gestation in comparison to those who had discontinued DMDs more than 4 weeks prior to conception. There was no statistical significant difference in fetal abnormality, abortion, preterm labor, and instrumental delivery. Nonetheless, it generally has been advised to stop INF-β at least 4-8 weeks before conception, especially in a stable MS patient. Conception is occasionally not successful in the predicted period and its delay may enhance the risk for relapse. Hence, because of low risk of INF-β according to anecdotal case series and clinical experience, some physicians prefer to continue INF-β until conception- without window period- especially in patients with active disease and high relapse rate in past years.^33^

*Glatiramer acetate (GA):* Based on animal studies (rats and rabbits) and analyzing human case reports GA safety in pregnancy has been categorized as level of B.^31^ However, GA should be considered during pregnancy only in high risk patients who want to be pregnant despite active disease or in a patient with relapse during pregnancy. If discontinuation of the drug is considered, no window period might be needed.^33^

*Natalizumab:* Experimental studies of guinea pig and primates elucidated that fetal exposure to natalizumab is associated with decreased survival and hematologic disorders in fetus.^36^ Five abortions and one hexadactyly have been reported in prospective evaluation of 35 women who exposed to natalizumab.^36^ Recent analyzing of 300 pregnant women who received natalizumab disclosed no significant side effects. Nonetheless, natalizumab has been categorized as level of C and general consensus is discontinuation of natalizumab about 3 months before conception.^33^

*Fingolimod:* It is the first FDA-approved oral treatment for MS.^37^ Although, it has agonistic effect on sphingosine-1 phosphate receptors (S1PR); by downregulation of S1PR, behaves as a functional antagonist of S1PRs.^38^^-^^39^ S1PRs are involved in fetal angiogenesis.^31^^,^^40^ According to potential teratogenicity in pregnant rats and other experimental results, it has been classified as level C.^31^ Until documentation of its safety in pregnant women, it is recommended that during and two months after discontinuation of fingolimod, a reliable contraception should be considered.^31^ Of note, at least two months is necessary for complete elimination of drug from the body.

*Mitoxantrone:* Mitoxantrone as most of other antineoplastic agents is contraindicated in pregnancy (characterized as level D) and lactation.^31^

*Teriflunomide: *By inhibiting pyrimidine synthetase, teriflunomide acts as a potential teratogen during embryogenesis as was documented by animal studies,^41^^-^^43^ so that it is contraindicated during pregnancy and lactation, designated as class X by the FDA.^31^ It has prolonged half-life (about 10-12 days) and may stay in the patient’s body about 8 months to 2 years after drug cessation.^44^ It is advised that teriflunomide not be used in women with unreliable contraception method. A pregnancy test should be done before starting the drug. Women, who want to become pregnant, must discontinue teriflunomide 8 months to 2 years before pregnancy and should not try to conceive a child until confirming drug elimination by blood tests.^33^^,^^44^ Cholestyramine and activated charcoal are advised to use due to their enhancing effect on drug elimination from the body. Besides, because of considerable semen concentration of teriflunomide, above protocol is advised as well to men who received this drug.^45^

***5.    Does elective abortion is indicated if pregnancy happens during consumption of DMDs?***

In unprogrammed pregnancies, DMDs other than GA, which could be continued according to disease activity, should be stopped as soon as possible.^43^^-^^44^ But, even in the case of teriflunomide, incidental exposure to DMDs is not an indication for elective abortion.^31^ In that case immediate drug stopping and augmented elimination protocol by cholestyramine or activated charcoal should be considered.^31^^, ^^45^

***6.    What is the preferred protocol for relapse treatment in pregnancy?***

It appears that short term (3-5 days) high-dose steroids is safe in pregnancy especially in the second and third trimester so that is advised by experts as a suitable treatment for MS attack during pregnancy. However, because of unavailable conclusive data about prednisolone and methylprednisolone; they have been designated as group C.^33^ Other than dexamethasone and Betamethasone; corticosteroids concentration in fetal circulation is about one tenth of maternal level due to metabolization to a less active metabolite by placental synctiotrophoblasts.^46^ So, their potential fetal malformation is minimal.^46^ They minimally increased risk of cleft lip, fetal adrenal suppression, transient neonatal leukocytosis (dexamethasone), fetal immunosuppression (methylprednisolone), and preterm premature rupture of membranes.^46^^-^^48^

Although, intravenous gamma globulin (IVIG) is classified as category C medication for pregnancy; it has been shown to be a suitable option for treating intractable MS relapse in gestational period.^49^ Its safety and tolerability during pregnancy elucidated based on the administration in other diseases. However, physicians should be aware of the possible adverse effects such as cerebral vein thrombosis which its risk is increased in pregnancy.^50^

***7.    Does maternal MS affect gestational outcome?***

There are some studies which suggest that maternal MS could be associated with a small but statistically significant increase in the risk of intrauterine growth retardation and preterm birth compared with women without MS.^51^^,^^52^ According to some other studies, there was no increased number of pregnancy or delivery complications, stillbirths, ectopic pregnancies, children with birth defects, preterm births, or spontaneous abortions in MS patients.^53^ However all evidence regarding these complications of MS on pregnancy is not entirely consistent.^54^

***8.    Is MS disease an indication for cesarean section delivery?***

Decision about mode of delivery usually is dependent on obstetrical indications rather than MS disabilities. However, it may be reasonable to consider the cesarean section or vacuum extraction for MS patient who are unable to push in the second stage of labor because of severe pelvic floor muscles weakness.^31^

***9.    Which mode of anesthesia is preferred in MS patients?***

At present, there is controversy about risk and benefits of surgery and mode of anesthesia in MS patients and their first relatives. National Multiple Sclerosis Society (NMSS) announced that surgery itself is not a risk for MS exacerbation; instead complications such as fever and infection can aggravate MS symptoms.^55^^-^^56^^,^^58^ Nowadays, there is general consensus that elective surgery is not contraindicated in MS patients and can be done as safe as normal population. Nonetheless, potential need for mechanical ventilation and prolonged respiratory support should be considered for severely disabled patient and who have respiratory problem.^57^^,^^58^ Despite lack of any acceptable evidence about the risk of surgery and either method of anesthesia, obtaining well-documented informed consent from patients even in the emergency setting is recommended.^59^^,^^60^

Based on anecdotal cases, previously assumed that spinal anesthesia intriguingly increases relapse rate in MS patient compared to general anesthesia.^61^^,^^62^ It was considered on the basis of a hypothesis that local anesthetics are neurotoxic for demyelinated white matter. Besides, it has been reported that high dose of anesthetics especially bupivcaine, and intrathecal (not epidural) anesthesia are independent risk factors for postoperative MS relapse.^55^^,^^63^ Recent data did not support this hypothesis. It appears to be a mathematical bias rather than the exact risk.^62^ Epidural anesthesia is safe and even is the preferential mode of anesthesia during labor especially in patients with extensive spinal cord lesion above T6, the level of splanchnic outflow, who are at risk for autonomic dysreﬂexia.^64^ Unlike epidural anesthesia, it may be reasonable not to advise intrathecal anesthesia until confirming its safety in well-designed studies.

Regarding anesthetics drugs, there is no contraindication or recommendation for use of especial drug in MS patients.^58^ However, physicians should be aware of increased risk of hyperkalemia following use of succinylcholine in MS especially in severely disabled patients with atrophied muscles. Severe muscle weakness and atrophy predispose patients to exaggerated paralytic effect of non-depolarizing muscle relaxant.^65^

Another issue that should be considered is body temperature. As little as 1C increase of body temperature is enough for aggravating patient’s symptoms. Therefore, anesthesia providers should carefully monitor body temperature and manage hyperthermia*.*^58^

***10.    How to manage postpartum period in patients with MS?***

As aforementioned above, approximately 33% of MS patients are at risk of relapse in postpartum period.^30^ Lactation induced amenorrhea has been speculated by some investigators to be protective with a 4-fold postpartum relapse reduction rate via downregulation of TNF-γ-producing CD4^+^ cells.^30^^,^^65^ Accordingly, at least 2 months of breastfeeding in postpartum period before starting DMDs is advised by some researchers. 

There is likely to forbid lactation and begin DMDs immediately after the delivery in high risk patients.^33^ Therapeutic effects of DMDs may be postponed for several weeks so that a rapid onset protective protocol such as high-dose steroid in early post partum period may be a reasonable option. Relapse reduction effect of steroids prolongs for approximately 4 weeks.^66^ In patients with active disease who want to continue lactation, monthly administration of 1 gram methylprednisolone is recommended by some researchers for a few weeks as disease modifying regimen. Of note, breastfeeding should be discontinued for 24 hours.^67^

Haas et al.^68^ showed that IVIG administration in women breastfed more than 3 months reduces the relapse rate about 33% if it is administered immediately after the delivery which was compatible with observations of Achiron et al.^69^ According to above evidence, some experts in MS recommend IVIG for the treatment of MS relapse during pregnancy, steroid resistant relapses, and as a safe DMD immediately after delivery.^68^^-^^73^ IVIG has been better to be prescribed at 1 (immediately after delivery), 6, and 12 weeks after the post partum period. 

Data regarding DMDs in lactation is not conclusive in order that until confirmation of DMDs safety in lactation, breastfeeding is recommended to discontinue if DMDs are mandatory to be started. It should be considered that GA is contraindicated during lactation (L3) which is similar to INF-β. 

***11.    What are imaging considerations in pregnancy and lactation period?***

Although, most studies investigated the MRI safety in pregnancy disclosed no serious side effect; teratogenicity and acoustic damage are two important fetal concerns which became topic of interest of recent investigations.^74^^-^^76^ Potential risk of acoustic damage to the fetus was studied by Baker et al.^77^ They speculated that the conventional MRI does not increase the risk of acoustic damage, but it is rather a theoretical concern.^78^ In general, MRI appears safe in human pregnancy especially after the first trimester and with 1.5 Tesla or less machines. 

Animal studies realized that gadolinium is potentially teratogen especially when it is employed in high dose.^78^ It is labeled as category C drug by the FDA. According to American Colleague of Radiology (ACR) Guideline, intravenous gadolinium is contraindicated in pregnancy especially in the first trimester, unless there is an absolutely essential indication based on maternal concerns.^79^ Nonetheless, risks and benefits should be discussed with patient before the drug administration.

MRI is not contraindicated in breastfeeding. However, 24 hours after administration of gadolinium, mothers preferentially should discontinue breastfeeding and discard the expressed milk of this period.^79^^,^^80^

***12.    Are contraception and in-vitro fertilization allowed in MS patients?***

Available guidelines did not consider any contraindication for conventional contraceptive methods in MS patients.^33^ Of interest, oral contraceptive (OCP) theoretically seems to have beneficial effects on MS course. However, no clinical trial supported this hypothesis with conventional dose of estrogen in OCPs.^81^ However, physicians should be aware of probable decreased contraceptive effect of OCPs due to concomitant use of enzyme-inducing drugs such as carbamazepin, modafinil, dantrolene on one side, and increased risk of hypercoagulability state in immobile MS patients on the other side.^33^^,^^81^

Foucher et al.^82^ in a retrospective analysis of in vitro fertilization (IVF) in six MS patients showed that gonadotropin-releasing hormone (GnRH) agonists which are used for ovulation induction in infertility management intriguingly enhance the relapse rate in MS patients. Of note, because of hormonal alteration similar to postpartum period, IVF failure has been reported to increase relapse rate in MS patients. GnRH antagonists had no effect on MS course.^82^

## Conclusion

Overall, pregnancy not only has not harmful effect on MS course, but it also seems to be beneficial. Nonetheless, physicians should be aware of maternal and fetal concerns in MS disease and manage every patient with considering her/his especial condition. There is general consensus that because of probable fetal side effects, DMDs be discontinued before conception with considering a window period (based on type of drug). However, GA appears to be a reasonable option during pregnancy in patients with active disease. Relapse can be treated with a short course of methylprednisolone pulse or IVIG (in intractable attacks). Likewise, IVIG is recommended by some experts as a disease modifying agent in postpartum immediately after delivery especially in patients with active disease. All DMDs are contraindicated in lactation. Thereupon, breastfeeding should be discontinued if DMDs are mandatory to be started. At the end, recognizing the precise mechanism of pregnancy-induced immunomodulation in MS as an interesting area for future investigations could provide insight into better understanding of disease pathogenesis and designing novel therapeutic strategies
